# Natural Language Processing for Automated Classification of Cleft and Craniofacial Procedures From Operative Notes: Model Development and Feasibility Study

**DOI:** 10.2196/87133

**Published:** 2026-05-11

**Authors:** Meredith Cox, Elaine Lin, Nicholas Oleck, Carlee Jones, Neill Y Li, Suhail K Mithani, Alexander C Allori

**Affiliations:** 1 Division of Plastic, Oral, and Maxillofacial Surgery, Duke University Hospital, 2301 Erwin Road, Durham, NC, 27710, United States, 1 919-668-3110; 2 Department of Orthopaedic Surgery, Duke University Hospital, Durham, NC, United States

**Keywords:** machine learning, natural language processing, cleft lip, cleft palate, craniofacial abnormalities

## Abstract

**Background:**

The accurate classification of operative notes is essential for surgical outcomes research; however, CPT code classification is notoriously nonspecific for many procedures. In such situations, the operative note (or “dictation”) must be reviewed manually, a process that is labor-intensive and unsustainable. Natural language processing demonstrates tremendous potential for improving the efficiency and accuracy of procedure classification from unstructured operative notes. To date, it remains unexplored whether natural language processing can reliably differentiate between complex, multicomponent procedures, such as those involved in the care of cleft lip or palate and craniofacial anomalies.

**Objective:**

This study aims to develop and evaluate a machine learning framework for the automated classification of operative notes for cleft and craniofacial procedures.

**Methods:**

This single-institution, retrospective observational study used operative notes from patients undergoing cleft and craniofacial procedures at a single academic medical center from 2016 to 2024. Each note in the database had been manually classified previously. Notes were preprocessed and vectorized using term frequency-inverse document frequency. A One-vs-Rest classification framework with random forest as the base classifier was developed to categorize procedures at 3 levels: primary procedure type (cleft lip repair, alveolar bone grafting, cleft palate repair, velopharyngeal insufficiency correction, rhinoplasty, and other), procedural subtype (primary vs revision), and specific surgical technique used (eg, Fisher, Mulliken, or rotation-advancement technique for cleft lip repair). Each hierarchical level was developed and evaluated using cross-validation. To improve procedural subtype classification for classes with few samples, synthetic notes were added to the dataset. Area under the receiver operating characteristic curve (AUC), an area under the precision-recall curve, micro- and macro-averaged *F*_1_-scores, and Hamming loss were used to assess model performance.

**Results:**

The dataset comprised 630 operative notes from 311 pediatric patients undergoing cleft and craniofacial procedures between 2016 and 2024, with a mean age of 3.75 (range 0‐19) years. The primary classification model achieved strong performance in distinguishing procedure types with an AUC of 0.93 (SD 0.04), area under the precision-recall curve of 0.84 (SD 0.05), micro-averaged *F*_1_-score of 0.88 (SD 0.02), a macro-averaged *F*_1_-score of 0.84 (SD 0.03), and a Hamming loss of 0.04 (SD 0.01). Secondary classifiers achieved AUC scores of 1.0 (SD 0.00) for cleft lip revision classification but failed to discriminate between alveolar bone grafting primary and revision procedures (AUC 0.49, SD 0.02). Tertiary classifiers for surgical technique identification showed AUC scores of 0.88 (SD 0.03), 0.89 (SD 0.03), and 0.89 (SD 0.09) for cleft lip, cleft palate, and velopharyngeal insufficiency repair techniques, respectively.

**Conclusions:**

This pilot study demonstrates that machine learning approaches can automate the classification of pediatric craniofacial operative notes across multiple levels of procedural detail. The implementation of such systems could significantly reduce the administrative burden related to surgical research, operations, and quality improvement.

## Introduction

Modern health care generates an overwhelming amount of data, ranging from structured information, such as demographic data and laboratory values, to unstructured content, such as clinical notes and radiographic images. While structured data may be readily analyzed using traditional statistical analysis and machine learning approaches for tasks such as disease risk stratification and outcome prediction [1-3], the wealth of clinical information contained in unstructured text data has remained largely untapped due to analytical challenges [4]. In the past, the analysis of unstructured data required manual chart review to extract desired elements and structure them in a spreadsheet or database for later use—a process that is labor-intensive, slow, costly, and subject to human error.

One example of where manual review of text data has been critically important is the review of operative notes (or “dictations”) that are recorded by a surgeon after a procedure has been completed. These notes contain essential information on the indication for the procedure (why it was performed), context or historical details about the patient leading to the decision, a description of the technical steps involved in the procedure, a summary of operative findings, and many other details. While standard coding systems, such as Current Procedural Terminology (CPT), can indicate with some generality the type of procedure performed, these codes are notoriously imprecise at representing the critical details about procedures, due to limitations in the codes themselves and variability in coding practices by surgeons and institutions [5]. As a brief example, consider that primary cleft palate repair may be represented by CPT codes 42200, 42205, 42210, and 42235. Revision palatoplasty is ideally coded as 42215, but it may also be coded using the same 42200 and 42205 as a primary palatal repair. Oronasal fistula repair is often coded using 30600, except that it may also be coded using revision palatoplasty codes 42215 or 42235. An alveolar bone graft procedure has no specific code of its own, so it is often coded using the palatoplasty code 42210. There is nothing in the code itself that provides information about which procedure was performed, why it was performed, how it was performed, or what happened during the time it was being performed. It is not the CPT coding system that is at fault, as it was designed for billing purposes and functions adequately in that regard. The problem lies in the fact that researchers have relied upon the CPT code (a structured data field) because it is more easily accessible and analyzable than manually extracting the necessary information from operative notes. Unstructured data have historically been inaccessible, impenetrable, or impractical.

Fortunately, recent advances in natural language processing (NLP) and machine learning have demonstrated promising results in the classification of procedures across various specialties [6-10]. These technologies offer potential solutions using machine learning for automatic procedural classification in fields such as orthopedics, general surgery, neurosurgery, ophthalmology, and anesthesiology, enabling more accurate and efficient clinical data retrieval and analysis. However, the application of these techniques to more complex procedures, such as cleft and pediatric craniofacial procedures, remains largely unexplored [11,12]. A child with a cleft lip or palate or another craniofacial anomaly undergoes operative and nonoperative care, typically coordinated by a multidisciplinary team, in stages spanning 2 decades of the child’s life, from birth through young adulthood. For children with some types of clefts, it is typical to undergo 4 to 6 cleft-related procedures in their lifetimes, and sadly, it is not uncommon to encounter patients who have undergone approximately 20 procedures. To decrease the number of anesthetic events for patients, many procedures are combined under the same anesthetic. The surgical techniques used vary considerably based on cleft phenotype, context, and surgeon preference. Such complexity in timing, sequence or staging, type of procedure, technique, coordination of procedures, and so forth makes cleft care the perfect testing ground for the refinement of NLP techniques for the extraction of critical details from the operative note.

In this pilot study, we developed and evaluated a machine learning framework for the automated classification of operative notes across multiple procedure types and subtypes related to cleft care. To address the “black box” nature of machine learning models, we used feature importance analysis to identify the specific textual elements that drive classification decisions. This work has the potential to enhance clinical documentation efficiency, facilitate large-scale quality improvement initiatives, and support comprehensive outcomes research in surgery.

## Methods

### Data Source

For this study, we extracted operative notes from all patients who underwent cleft and craniofacial procedures at a single institution from 2016 to 2024, comprising 656 operative notes from 312 patients. Operative notes were dictated by the primary surgeon immediately following the completion of the procedure. These notes were stored contemporaneously within the electronic medical record and were periodically aggregated into a condition-specific database as part of the guidelines recommended by the “Standard Set of Outcome Metrics for the Comprehensive Appraisal of Cleft Care” of the ICHOM (International Consortium for Health Outcomes Measurement) [13] and the operational protocol of the Allied Cleft & Craniofacial Quality-Improvement and Research Network (ACCQUIREnet) [14], of which our institution is a member. Within the condition-specific databases, procedures are classified using the ACCQUIREnet Common Data Model. At our institution, this labeling has been performed by an experienced annotator (CJ), a speech pathologist (CCC-SLP), and the Team Coordinator of an American Cleft Palate Craniofacial Association–approved Cleft & Craniofacial Team since 2018. Any uncertainty in procedural classification was resolved by a senior surgeon (ACA) with over 20 years of clinical experience and the director of ACCQUIREnet. Procedural classification was performed for case type (eg, cleft lip repair, cleft palate repair, alveolar bone grafting), subtype or stage (primary repair or revision), and technique. Where operative notes contained the documentation of multiple procedures done in coordination (such as cleft lip repair being done with tip rhinoplasty, or cleft palate revision being done with sphincteroplasty for speech), each subprocedure was classified separately.

### Generation of Synthetic Notes to Augment Dataset

Some procedures were severely under-represented in our dataset. For these procedures, the dataset was augmented using synthetic notes generated by a large language model (LLM). To generate these synthetic operative notes, we used GPT-4o (OpenAI) [15] with a multishot prompting strategy guided by manually anonymized, real operative notes. Example notes were drawn from a separate Duke cleft and craniofacial dataset collected outside the study period to guide the model in reproducing procedure-specific structure and terminology without introducing data leakage. To guide overall structure, 3 representative examples from an internal dataset not used in model development were provided for each procedure while encouraging variation in operative technique and language. Using this approach, we generated synthetic operative notes for cleft lip major revision, cleft lip minor revision, alveolar bone grafting revision, and velopharyngeal insufficiency repair by cleft palate revision with counts matched to the corresponding real-note datasets.

A senior cleft or craniofacial surgeon (ACA) reviewed the synthetic notes to appraise content validity. All notes were rated as being satisfactory in quality and content, similar to what would be found in clinical practice. Criticisms included unnecessary verbosity, slight disorganization in the operative sequence, limited detail in some parts of the operation, and lack of variation with regard to technical complexity or intraoperative events (some cases are harder than others). Notwithstanding, the limitations were actually viewed as advantageous to this project, as they might better simulate the varying quality of notes “in the wild”—for example, what a junior resident or hurried attending surgeon might have dictated.

Synthetic notes were used solely for model development and not for evaluation.

### Preprocessing

Operative notes often contain content such as patient history, procedure indications, or other metadata. To isolate clinically relevant content, a custom Python script (version 3.11.9) extracted specific sections of interest (eg, “indications,” “operative findings,” and “operative details”) from the body of each note. This script identified and extracted content preceded by section headers, detected all candidate headers, and then split the note into segments at each detected header. Because the base template is auto-generated by the electronic health record, headings are consistently present; however, certain sections can be overwritten by the surgeon (dictated or typed as free text). The user specified desired content (eg, “operative details”) as input, and the script used a combination of exact and fuzzy string matching to identify synonymous headers (eg, “operative technique” or “procedure description”). To improve header recognition consistency across notes, the script optionally supported training for header deduplication using the dedupe library (version 3.0.3), which learns common aliases from the dataset and creates a mapping dictionary [16]. For this process, a separate set of operative notes was used—that is, notes that were not included in the dataset for subsequent vectorization and analysis. Using this mapping, operative notes were split into sections, which were saved for downstream analysis.

The procedure notes were preprocessed to prepare them for NLP analysis. The preprocessing step is crucial for standardizing the text data and reducing noise in subsequent analysis. The medium-sized English-language model from the spaCy NLP library was used for text processing (version 3.8.4). Each operative note underwent tokenization, lemmatization, and filtering to remove punctuation, stop words (such as “the,” “a,” “and,” “in,” “of,” and “is”), and nonalphabetic characters. Clinically relevant negation terms (such as “no,” “not,” and “without”) were preserved.

### Classifier Model Development

The classification framework used a One-vs-Rest (OvR) strategy with random forest as the base classifier. This approach was selected for several compelling reasons. First, OvR decomposes the complex multilabel problem into multiple binary classification problems (one for each procedure type), significantly reducing computational complexity while maintaining high performance. Second, this methodology preserves the interpretability of the model’s predictions. Third, OvR classifiers handle class imbalance effectively when paired with appropriate weighting, which was particularly important given the uneven distribution of procedure types in the dataset. Class weighting of the random forest was used to address dataset imbalance, which penalizes the misclassification of minority classes more heavily during the training process. Given the small dataset size of 630 notes, deep learning methods were not likely to be optimal for the task, based on results from prior studies examining sample size requirements for medical NLP classification tasks with deep learning [17,18].

**Figure 1.**
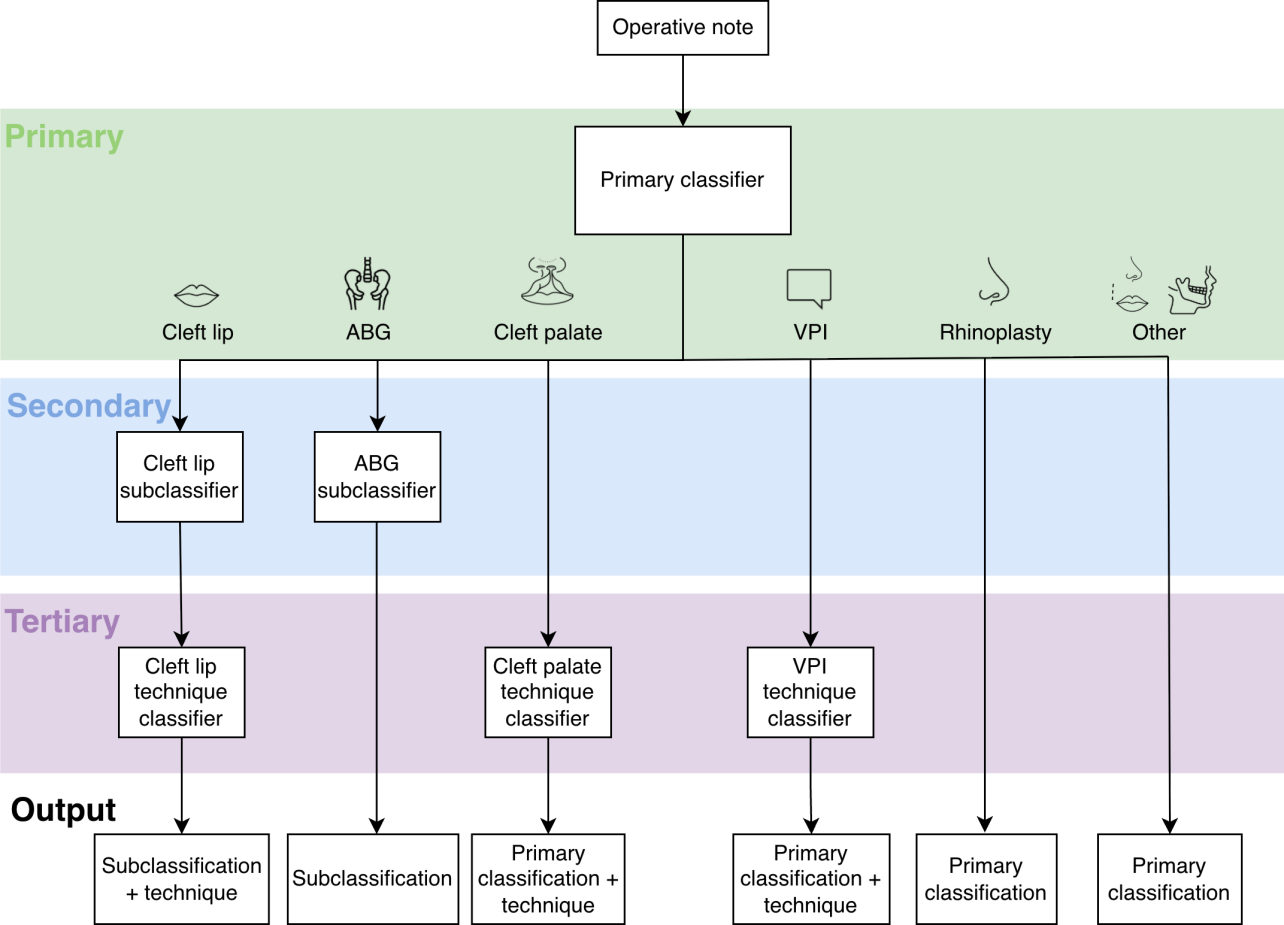
Classification schematic. A primary classifier categorizes an operative note by primary procedure (cleft lip, alveolar bone grafting [ABG], cleft palate, velopharyngeal insufficiency [VPI], or rhinoplasty). A secondary classifier further categorizes cleft lip and ABG procedures into primary and revision procedures. A tertiary classifier categorizes cleft lip, cleft palate, and VPI procedures by technique.

The overall architectural design implements a hierarchical classification framework with 3 levels: primary procedure-type classification, secondary subtype classification for applicable procedures, and tertiary technique classification where sufficient data exist. This hierarchical propagation yields a comprehensive procedural characterization with progressive granularity. The classification architecture is illustrated in Figure 1.

For the primary classifier, we used patient-level grouped 5-fold cross-validation to ensure all notes from each patient remained in either the training or test set, preventing potential information leakage through patient-specific documentation patterns. For secondary and tertiary classifiers, insufficient sample sizes (particularly for minority classes) precluded patient-level grouping while maintaining adequate class representation in each fold. Note-level grouping with 3-fold cross-validation was used for secondary and tertiary classifiers to ensure sufficient representation of minority classes within each fold.

Term frequency–inverse document frequency (TF-IDF) vectorization converted the preprocessed text into a format suitable for machine learning analysis. This technique weights terms based on their frequency within individual documents relative to their occurrence across the entire corpus, effectively capturing the relative importance of specific terms within each operative note. TF-IDF was used for text vectorization due to its interpretability, allowing individual words to be linked to model predictions. Explainability leads to greater physician trust in medical artificial intelligence tools, and understanding how models are making decisions is important prior to incorporating them into clinical and research workflows [19,20]. We also experimented with word embeddings, including Word2Vec and bidirectional encoder representations from transformers, but these approaches yielded similar predictive performance on our tasks. Thus, TF-IDF offered a practical balance of performance and interpretability. To better capture multiterm clinical concepts (such as “water” and “tight” signifying “water-tight closure” of the mucosa in cleft palate repair), as well as negation, we included unigrams and bigrams in our analysis. We also selected only the top 500 features for inclusion in our model. To prevent data leakage, the TF-IDF vectorizer and feature selection were fit exclusively on the training fold during each cross-validation iteration and subsequently applied to the held-out test fold. The resulting TF-IDF matrix served as the feature set for the classification model.

The decision threshold was chosen to optimize *F*_1_-scores, as the *F*_1_-score is a combination of precision and recall, both of which are helpful for different use cases, such as precision for screening cohorts and recall for registry automation. For each fold, the *F*_1_-score at multiple thresholds was calculated, and the optimal threshold was chosen. The average threshold across folds was selected for the final model.

### Primary Classification: Procedure Type

For primary classification, a multilabel classification model was developed to automatically categorize operative notes into 5 distinct procedure types common in craniofacial surgery: cleft lip repair, alveolar bone grafting (ABG), cleft palate repair, velopharyngeal insufficiency (VPI) correction, rhinoplasty, and other procedures. “Other” procedures include noncleft and craniofacial procedures, such as myringotomy and tympanostomy tube placement, as well as cleft and craniofacial procedures with only a small number of procedures recorded, that is, oronasal fistula repair (14 procedures) and orthognathic repositioning (6 procedures).

The model used multilabel classification, as a single operative note frequently described multiple procedure types performed during the same surgery, and it is important to identify combination cases. For example, it is common for a cleft lip repair to be performed alongside a limited-tip rhinoplasty. Unlike traditional multiclass classification, where each instance belongs to exactly one class, multilabel classification allows each operative note to be assigned to any number of relevant categories simultaneously, from zero to all 5 procedure types. This approach reflects the clinical reality of complex craniofacial surgeries, where multiple procedures are often performed during a single operation.

### Secondary Classification: Procedure Subtype or Stage

For secondary classification, OvR classifiers that were able to distinguish between subclassifications of procedures were developed. These classifiers were developed in isolation, given ground-truth primary classification. For example, given cleft lip repairs, the classifier could determine whether the procedure was a primary repair or revision. Classifiers like these were developed for cleft and craniofacial procedures in the dataset that had sufficient examples for testing (>1 in the test set). Procedures with enough samples for subclassification include cleft lip and ABG. Procedures such as cleft lip major and minor revisions, as well as revision ABG, had low counts of 4, 5, and 9, respectively. For these procedures, the dataset was augmented using synthetic notes generated by an LLM.

### Tertiary Classification: Technique

At the most granular level of our hierarchical framework, a classifier was developed that could distinguish between specific surgical techniques used within procedural subclassifications. This classifier was developed in isolation, given ground-truth primary and secondary classifications. In the context of cleft lip repairs, for instance, the tertiary classifier discriminates between the Fisher, modified rotation-advancement, Mulliken, and other techniques. For cleft palate repairs, the classifier distinguishes between von Langenbeck, Bardach, Veau-Wardill-Kilner, Furlow, Sommerlad, and other palatoplasty techniques. For VPI repairs, the classifier distinguishes between VPI repair by cleft palate revision or sphincteroplasty or pharyngoplasty. Synthetic note generation was used for notes describing VPI repair by cleft palate revision, for which there were only 6 examples.

### Model Evaluation

The performance of the classification model was assessed using multiple metrics. To evaluate the model’s discrimination ability, the area under the receiver operating characteristic curve (AUC) and the area under the precision-recall curve (AUPRC) were calculated for each procedure type individually. We also report a macro-average across all classes, which provides insight into the model’s performance regardless of class prevalence. The balance between precision and recall was assessed by the *F*_1_-score, reported as both micro and macro averages to account for potential class imbalances in the dataset. Additionally, the fraction of incorrectly predicted labels was assessed using Hamming Loss, which is particularly informative in multilabel classification scenarios where partial correctness is meaningful.

All data processing and modeling were implemented in Python 3.11, utilizing scikit-learn (version 1.5.1) for machine learning operations, pandas for data manipulation (version 2.2.3), matplotlib for visualization (version 3.9.2), and spaCy for natural language processing (version 3.8.4). We maintained a fixed random seed to ensure the reproducibility of results.

### Ethical Considerations

This work was performed under institutional review board approval (Pro00104806) for participation in the ACCQUIREnet, a multisite collaborative network. Participating sites in the ACCQUIREnet collect standardized outcomes data and variables that were defined according to the ICHOM’s Standard Set of Outcome Metrics for the Comprehensive Appraisal of Cleft Care. The ACCQUIREnet is registered with ClinicalTrials.gov as an observational cohort study (#NCT02702869). All adult participants and parents of minor participants provided consent for participation in the data-collection processes of the network. In this work, operative notes and case classifications were reviewed from our single institution (not the entire network). Data processing and analysis were guided by all institutional policies and regulations regarding the protection of personal information, privacy, and human rights.

## Results

### Dataset Characteristics

The condition-specific database contained 656 operative notes for analysis. Twenty-six notes could not be parsed to extract a procedure description, resulting in a final cohort of 630 notes from 311 patients. The exclusion of the 26 notes resulted in the complete removal of 1 patient from the cohort. Of the 26 notes that could not be parsed, 3 contained unrecognized headers, 8 had no identifiable header, and 15 had improperly formatted headers (eg, lowercase or indistinguishable from body text). These notes represented cleft and craniofacial procedures between 2016 and 2024. Patients had a mean age of 3.75 (SD 4.1, range 0‐19) years. In 184 instances, multiple procedures were performed during the same anesthetic event. In total, 958 procedures were documented across the 630 notes.

Procedures included the following: 100 cleft lip repair (primary, secondary major, and minor revision), 105 alveolar bone grafting (primary and secondary revision), 104 cleft palate repair, 32 velopharyngeal insufficiency correction (via cleft palate revision, sphincteroplasty, or pharyngoplasty), and 36 rhinoplasty (limited-tip). The 581 “Other” procedures included 191 (32.7%) auditory procedures (audiometry evoked-potential brain response testing, myringotomy and tympanostomy tube placement, and patch myringoplasty), 92 (15.8%) suture removal, 33 (5.8%) dental rehabilitation, 14 (2.4%) oronasal fistula repair, 6 (1%) cranial procedures (suturectomy, cranial vault expansion, and fronto-orbital advancement), 6 (1%) orthognathic repositioning, 5 (0.9%) gastrostomy, and 234 (40.3%) heterogeneous group of less common interventions spanning multiple specialties, including ophthalmologic (eg, strabismus surgery, cataract extraction), neurosurgical (eg, ventriculoperitoneal shunt placement and revision, Chiari decompression), orthopedic (eg, fracture fixation, spinal fusion), general surgical (eg, exploratory laparotomy, bowel resection), urologic (eg, cystourethroscopy), and cardiothoracic procedures (eg, congenital heart defect repair).

Procedure descriptions averaged 244 words (median 223 words), ranging from 5 to 809 words. The corpus contained 5175 unique tokens, with “patient,” “place,” and “suture” being the most frequent. The type-token ratio of 3.36% indicated substantial lexical repetition and limited vocabulary diversity across operative notes.

### Primary Classification Performance

**Figure 2.**
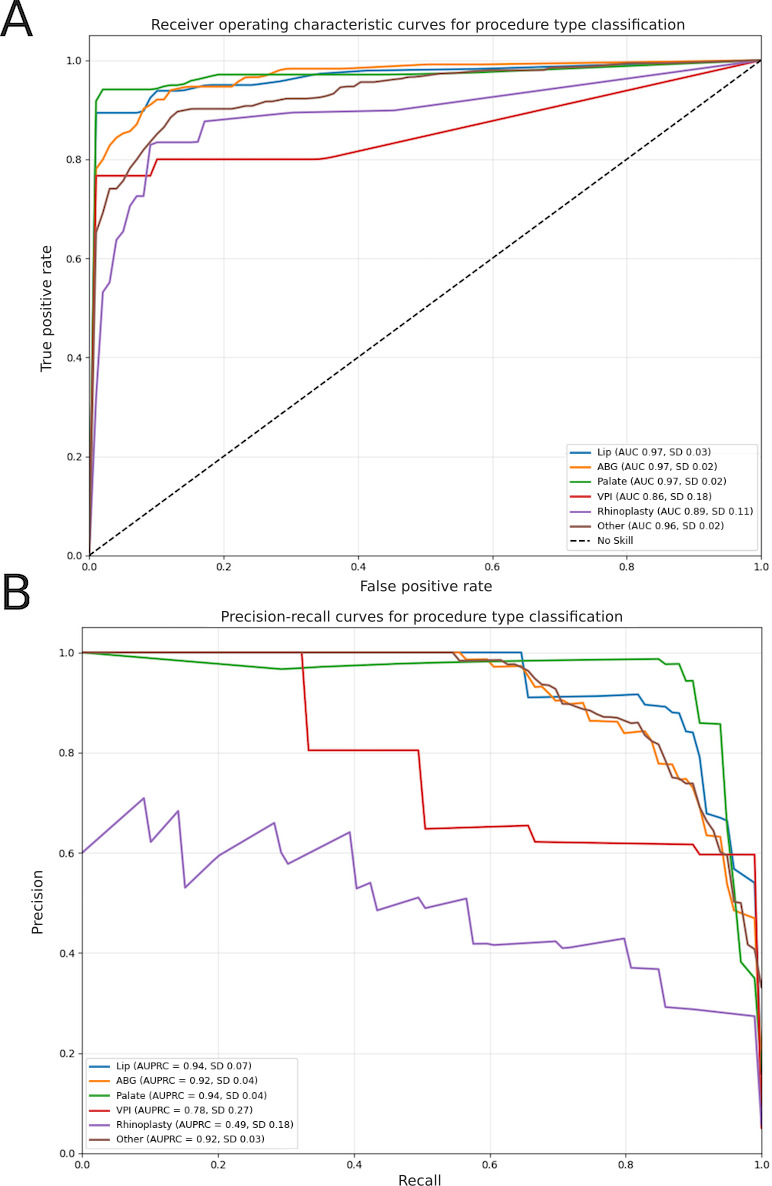
(A) Receiver operating characteristic curve of the primary classifier. The curve demonstrates the primary classifier’s performance for each procedure type as compared to a no-skill classifier. (B) Precision-recall curve of the primary classifier. ABG: alveolar bone grafting; AUC: area under the receiver operating characteristic curve; AUPRC: area under the precision-recall curve; VPI: velopharyngeal insufficiency.

The model demonstrated strong performance in distinguishing procedure types, achieving an AUC of 0.93 (SD 0.04), an AUPRC of 0.84 (SD 0.05), a micro-averaged *F*_1_-score of 0.88 (SD 0.02), a macro-averaged *F*_1_-score of 0.84 (SD 0.03), and a Hamming loss of 0.04 (SD 0.01). The AUC and AUPRC curves are shown in Figure 2.

### Secondary Subclassification Performance

**Figure 3.**
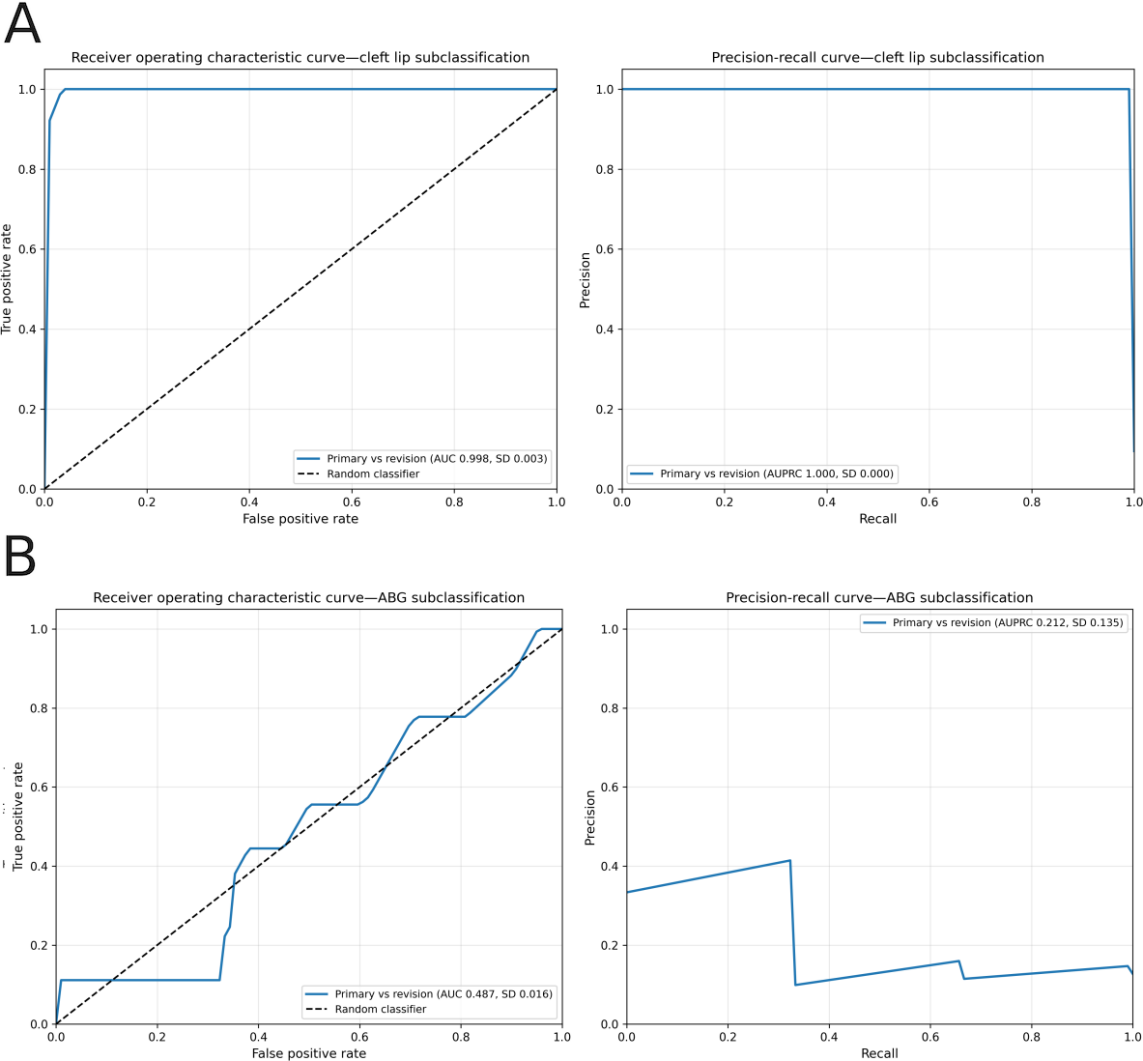
(A) Receiver operating characteristic and precision-recall curves for the cleft lip subclassification model, distinguishing primary versus revision procedures. (B) Receiver operating characteristic and precision-recall curves for the alveolar bone grafting (ABG) subclassification model, distinguishing primary versus revision procedures. AUC: area under the receiver operating characteristic curve; AUPRC: area under the precision-recall curve.

In distinguishing subclassifications, such as primary cleft lip repairs versus revisions, the model demonstrated variable performance. Performance was excellent for cleft lip revision, with an AUC score of 1.0 (SD 0.00), an AUPRC of 1.0 (SD 0.0), a micro-averaged *F*_1_-score of 0.96 (SD 0.02), a macro-averaged *F*_1_-score of 0.90 (SD 0.03), and a Hamming loss of 0.06 (SD 0.0) . In contrast, performance for ABG was poor, with an AUC of 0.49 (SD 0.02), an AUPRC of 0.21 (SD 0.14), a micro-averaged *F*_1_-score of 0.61 (SD 0.02), a macro-averaged *F*_1_-score of 0.41 (SD 0.05), and a Hamming loss of 0.39 (SD 0.02), indicating that the model failed to discriminate between primary and revision ABG procedures. Receiver-operating characteristic and precision-recall curves for secondary classifiers are shown in Figure 3. The slight discrepancy between AUC and *F*_1_-score for the cleft lip subclassification model reflects the use of a threshold averaged across folds, which may not be optimal for each individual fold. Cleft palate and rhinoplasty procedures had only 1 category represented in the data (primary cleft palate repair and limited-tip rhinoplasty), and therefore a classifier was not developed due to the lack of real examples for testing. VPI revision subclassification was not possible, as only the VPI technique was recorded in our dataset.

### Tertiary Technique Classification Performance

**Figure 4.**
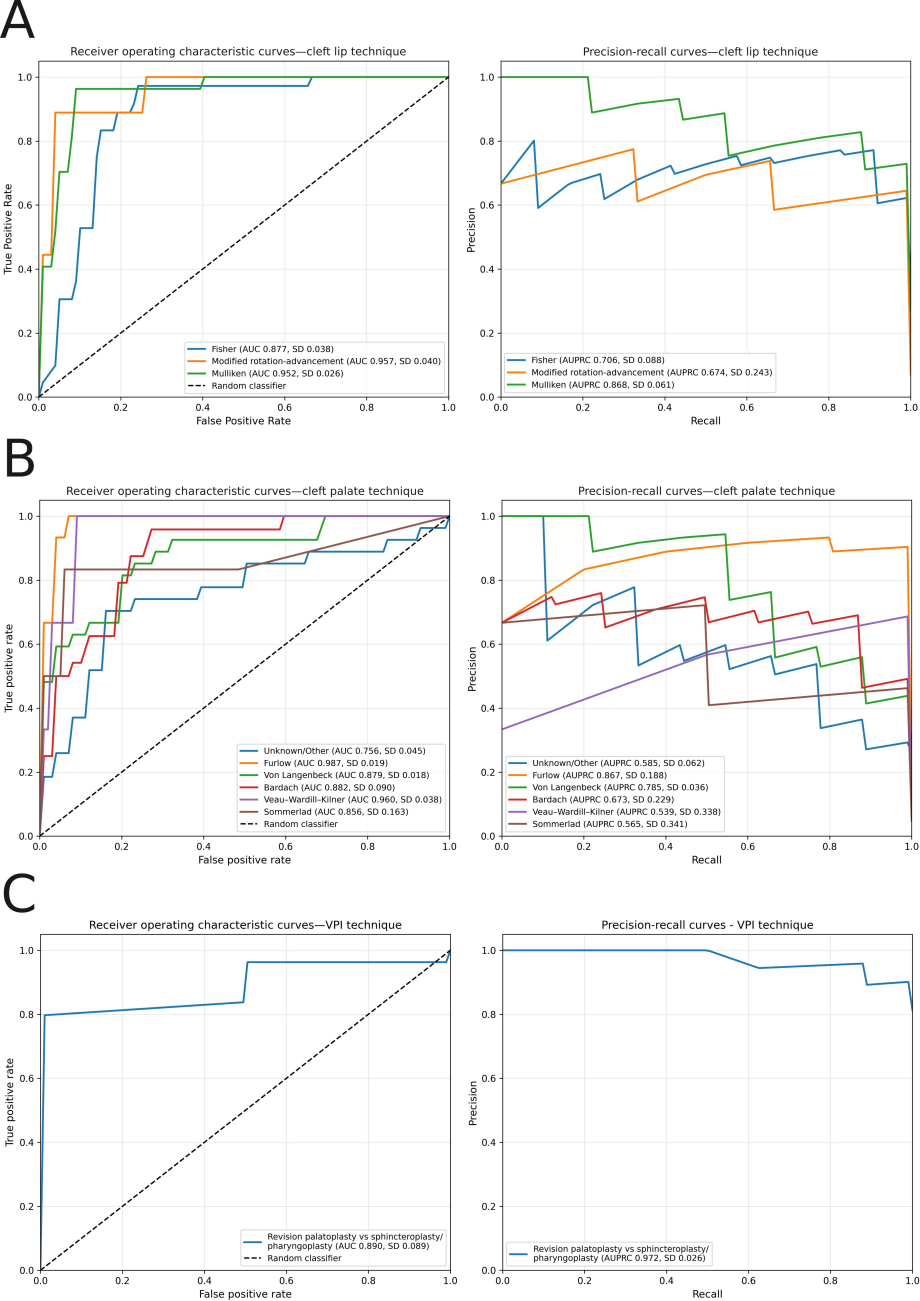
(A) Receiver operating characteristic and precision-recall curves for the cleft lip technique classifier. (B) Receiver operating characteristic and precision-recall curves for the cleft palate technique classifier. (C) Receiver operating characteristic and precision-recall curves for the velopharyngeal insufficiency technique classifier. AUC: area under the receiver operating characteristic curve; AUPRC: area under the precision-recall curve; VPI: velopharyngeal insufficiency.

The model for identifying procedure techniques also exhibited good performance, with AUCs ranging from 0.87 to 0.90. The full results are shown in Table 1. Briefly, the cleft lip technique classifier distinguishes between Fisher, modified rotation-advancement, Mulliken, and other techniques. The cleft palate technique classifier distinguishes between von Langenbeck, Bardach, Furlow, Veau-Wardill-Kilner, Sommerlad, and other techniques. The VPI technique classifier distinguishes between VPI repair by cleft palate revision and sphincteroplasty or pharyngoplasty. In our dataset, only 4 ABG procedures had associated techniques recorded, which was insufficient for developing a reliable classifier. Receiver-operating characteristic and precision-recall curves for tertiary classifiers are shown in Figure 4.

**Table 1.** Performance metrics for tertiary classifiers identifying specific surgical techniques^a^.

Technique	AUC^b^	AUPRC^c^	*F*_1_-score (micro)	*F*_1_-score (macro)	Hamming loss
Cleft lip, mean (SD)	0.88 (0.03)	0.72 (0.08)	0.77 (0.04)	0.77 (0.06)	0.22 (0.04)
Cleft palate, mean (SD)	0.89 (0.03)	0.67 (0.05)	0.65 (0.05)	0.65 (0.07)	0.36 (0.05)
VPI^d^, mean (SD)	0.89 (0.09)	0.97 (0.03)	0.81 (0.01)	0.45 (0.01)	0.19 (0.01)

a Metrics for categories with very small sample sizes (eg, velopharyngeal insufficiency repair by cleft palate revision, n=6) should be interpreted with caution, as limited representation in cross-validation may result in unstable estimates.

b AUC: area under the receiver operating characteristic curve.

c AUPRC: area under the precision-recall curve.

d VPI: elopharyngeal insufficiency.

### Feature Importance

**Figure 5.**
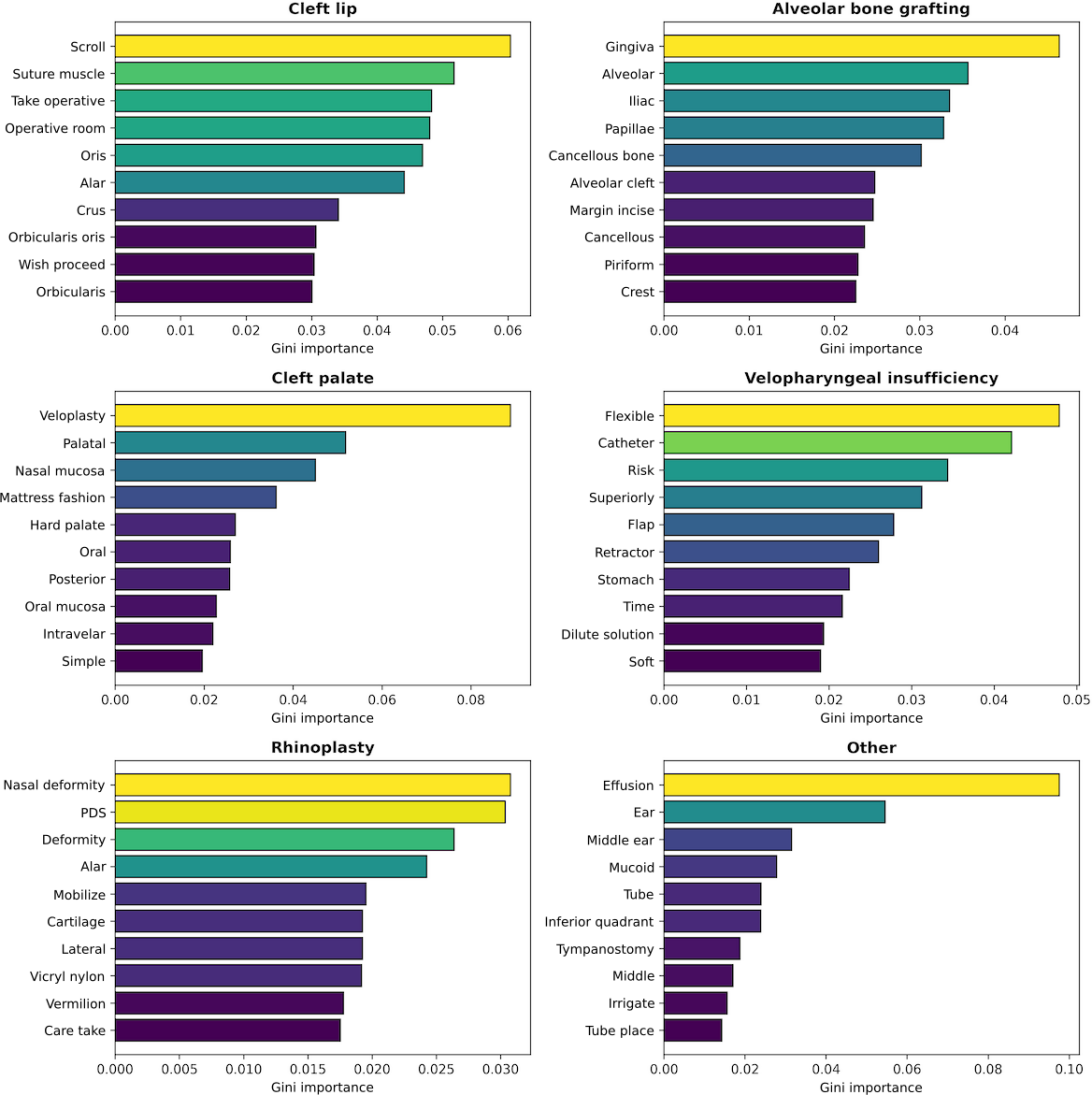
Primary classifier explanations. Top 10 most important term frequency-inverse document frequency (TF-IDF) features for each classification label, based on model coefficients. Bar color reflects normalized importance within each class.

Feature importance analysis identified key terms contributing to each procedure classification for the primary classifier. The top-ranked words reflected distinct operative terminology across categories. The relative importance values of the top 10 words for each procedure type are shown in Figure 5.

### Overcoming Limitations of CPT Coding

To further emphasize the need for cohort definition tools beyond CPT codes, we examined the CPT codes assigned to manually labeled VPI and ABG procedures, which are frequently miscoded due to the limited granularity of CPT coding. Tables2  3 illustrate the heterogeneity of CPT coding for VPI and ABG procedures, respectively, demonstrating the multiple ways identical or highly similar procedures are coded. In total, 32 VPI procedures and 105 ABG procedures were analyzed. Of note, these are two substantially different procedures, and yet the CPT codes overlap due to the generality of the codes, arbitrariness in selecting a code, and possibly even miscoding.

**Table 2.** Current Procedural Terminology (CPT) code variability across velopharyngeal insufficiency repair procedures (N=32).

CPT code	Description (if applicable)	Procedures, n (%)
42226	Lengthening of palate and pharyngeal flap	13 (40.6)
42950	Pharyngoplasty (plastic or reconstructive operation on pharynx)	7 (21.9)
42220	Palatoplasty for cleft palate; secondary lengthening procedure	4 (12.5)
42210	Palatoplasty for cleft palate, with closure of alveolar ridge; with bone graft to alveolar ridge (includes obtaining graft)	3 (9.4)
42215	Palatoplasty for cleft palate; attachment pharyngeal flap	2 (6.3)
42225	Palatoplasty for cleft palate; major revision	2 (6.3)
42200	Palatoplasty for cleft palate, soft and/or hard palate only	1 (3.1)

**Table 3.** Current Procedural Terminology (CPT) code variability across alveolar bone grafting procedures (N=105).

CPT code	Description (if applicable)	Procedures, n (%)
42210	Palatoplasty for cleft palate, with closure of alveolar ridge; with bone graft to alveolar ridge (includes obtaining graft)	68 (64.8)
40700	Plastic repair of cleft lip/nasal deformity; primary, partial or complete, unilateral	23 (21.9)
42200	Palatoplasty for cleft palate, soft and/or hard palate only	7 (6.7)
42205	Palatoplasty for cleft palate, with closure of alveolar ridge; soft tissue only	4 (3.8)
40701	Plastic repair of cleft lip/nasal deformity; primary bilateral, 1-stage procedure	2 (1.9)
21210	Graft, bone; nasal, maxillary, or malar areas (includes obtaining graft)	1 (1)

## Discussion

### Principal Findings

This study demonstrates that machine learning approaches can effectively classify operative notes in pediatric craniofacial surgery with high accuracy across multiple levels of granularity. The primary classification model achieved strong performance (AUC 0.93, SD 0.04) in distinguishing between 5 distinct procedure types common in craniofacial surgery. Secondary classifiers for procedure subclassifications demonstrated variable performance, with AUC scores of 1.0 (SD 0.00) for cleft lip revision and 0.49 (SD 0.02) for ABG. At the most granular level of surgical technique identification, our model maintained good discriminative ability (AUC 0.88, SD 0.03 to AUC 0.89, SD 0.09).

The model for ABG secondary subclassification demonstrated poor performance (AUC 0.49, SD 0.02). This is likely due to substantial overlap in operative and clinical language between primary and revision procedures. In many revision cases, surgeons perform repeat alveolar bone grafting using techniques similar to those used in primary repairs, leading to documentation that does not reliably distinguish initial from secondary procedures. As a result, the textual features available to the model may be insufficient to support accurate subclassification.

The VPI technique classifier demonstrated a notable discrepancy between the micro-averaged *F*_1_-score (0.81, SD 0.01) and the macro-averaged *F*_1_-score (0.45, SD 0.01), indicating a performance imbalance across technique categories. This gap suggests that while the model achieves high overall accuracy, it performs poorly on the minority technique. In our dataset, VPI repairs were categorized as either sphincteroplasty/pharyngoplasty or repair by cleft palate revision, with the latter representing only 6 real examples that required synthetic augmentation. The high micro-*F*_1_-score indicates strong performance on the more prevalent sphincteroplasty/pharyngoplasty category, which dominates the overall metric due to its larger sample size. In contrast, the low macro-*F*_1_-score reveals that the model struggles to correctly identify VPI repairs performed via cleft palate revision, likely due to substantial overlap in operative terminology between these approaches.

Additionally, AUC estimates for these very small classes (eg, VPI repair by cleft palate revision, n=6) are unstable and should be interpreted as exploratory until larger labeled datasets are available. The perfect AUC observed for the cleft lip secondary classifier should also be interpreted with caution. Unlike the primary classifier, this model used note-level rather than patient-level cross-validation due to limited sample size, meaning notes from the same patient or surgeon may have appeared in both training and test sets. The AUC of 1.0 may overestimate real-world generalizability.

This classification system has several important clinical implications. First, this study provides proof-of-concept that automated classification could substantially reduce the administrative burden on clinical teams. In many studies, traditional coding systems, such as CPT and *International Classification of Diseases* (*ICD*), codes are used for procedural and diagnostic classification. However, CPT coding has been shown to be inconsistent for craniofacial procedures due to inconsistent CPT codes that fail to capture complex techniques [21,22]. The pediatric craniofacial population presents unique documentation challenges due to the longitudinal nature of care, with patients often undergoing multiple staged procedures over many years. CPT codes frequently lack the granularity necessary to distinguish between subtle but clinically significant variations in surgical technique. For instance, CPT code 42200 encompasses multiple distinct approaches to cleft palate repair that have different implications for surgical planning and outcomes research. Additionally, procedures may be coded inaccurately—for example, the correction of VPI or ABG procedures may use a cleft palate repair CPT code. In the case of VPI, revision palatoplasty is one approach for correcting the disorder. In the case of ABG, no specific CPT code exists for bone grafting of the alveolar cleft, so it is conventional to use a “close-enough” CPT code for palatoplasty with bone grafting. The consequence of this “real-world” coding practice has been that manual review was needed to accurately classify complex procedure types. Of course, manual review and classification of operative notes are time-consuming and subject to human error. The substantial variability in CPT coding observed for both VPI and ABG procedures underscores the limitations of billing-based classification systems and highlights the added value of NLP-derived tertiary classifications for capturing clinically meaningful procedural detail. A major goal of this project was to provide an accurate, reliable, efficient, and sustainable method for automating case classification from the unstructured text data of the operative note, allowing for more accurate and efficient cohort identification.

Second, the analysis of feature importance provides insight into the distinctive language patterns that characterize different craniofacial procedures. The terms most strongly associated with each procedure type align with the clinical understanding of these operations, suggesting that the model has learned clinically relevant patterns. This interpretability is crucial for clinical adoption, as it allows surgeons to understand and validate the model’s decision-making process.

### Comparison to Prior Work

While prior work applying NLP to operative notes in cleft and craniofacial surgery is limited, related efforts in surgical subspecialties and clinical text classification provide useful points of comparison. A growing body of literature has demonstrated the potential of NLP for classifying surgical procedures based on operative notes, with many studies focusing on predicting CPT codes for billing purposes in fields such as general surgery [23], breast surgery [10], spine surgery [24,25], and pathology [26]. Recently, LLMs have been evaluated for CPT coding in craniofacial surgery given operative notes [27]. However, the accuracies of all 5 models tested ranged between 20% and 40%. These low accuracies suggest that general-purpose LLMs may be poorly suited for granular CPT code classification in highly specialized surgical domains, particularly when models are not trained on domain-specific operative language. Similar findings have been reported in other studies using generalized LLMs, which demonstrate low accuracy in classifying surgical procedures [8,28,29] and CPT modifiers in craniofacial operative notes [30]. Collectively, this work indicates that procedure classification may require models trained on specialized operative notes rather than relying on generalized LLMs alone.

Our work addresses this challenge within cleft and craniofacial surgery—a specialty characterized by complex, multicomponent procedures and longitudinal care spanning years of development—where the precise identification of surgical techniques is crucial for patient outcomes and clinical research. The *hierarchical* approach to classification, moving from procedure *type* (eg, cleft lip repair) to *subclassification* (eg, subtype or stage, such as primary vs revision) to *specific technique* (eg, Fisher anatomic subunit technique), represents a more nuanced and clinically relevant analysis than most previous studies, which typically focus on primary procedure identification or CPT code assignment only. Moreover, our approach does not treat procedural coding as the end goal. Rather, CPT codes serve primarily as a point of comparison. Instead, the proposed model enables more efficient and accurate cohort identification for outcomes reporting and surgical research by capturing both procedure type and detailed operative technique.

Another study highlights the development of a tool, ChartSweep, to automate chart review, which can be used for cohort identification [31]. The study highlights the significant amount of time spent on retrospective chart review compared to an automated tool (8 minutes per patient with manual chart review vs 0.3 minutes per patient with the automated tool for the identification of a radiofrequency ablation cohort). These findings underscore the inefficiency of manual chart review in plastic surgery research. Similar to ChartSweep, our study aims to reduce this burden through automation; however, we specifically address the challenge of classifying cleft and craniofacial procedures, where overlapping and reused operative terminology complicates automated extraction, an area not examined in the prior work.

We additionally apply a synthetic data generation technique to address class imbalance in rare procedure types, demonstrating a solution to a common challenge in specialty surgical fields with relatively low procedure volumes. Several studies have demonstrated the ability of LLMs to generate satisfactory medical notes. Such synthetic notes have been shown to be sufficiently realistic for downstream analytic tasks, with multiple studies proposing their use as a viable strategy to mitigate data scarcity [32-40]. In this context, our work extends the existing literature by demonstrating the use of synthetic data specifically for hierarchical surgical procedure classification, supporting its role in improving model performance for rare but clinically meaningful procedures.

### Limitations

First, our study used data from a single institution, potentially restricting the generalizability of our models to other clinical settings with different documentation practices. Operative notes can vary significantly in structure, terminology, and level of detail across institutions and individual surgeons. To address this concern, future studies should validate these models using multi-institutional data to assess their robustness across different documentation styles. This is planned as future work for the ACCQUIREnet.

Second, surgical notes often rely on templates for documentation efficiency. It is a concern that the model is overfitting to templated data. In this project, the templates for cleft and craniofacial operative notes have changed significantly in the time span encompassed by the study, and operative notes from multiple surgeons are present. This variability does diminish (but does not eliminate) the risk of overfitting. Feature importance analysis identified several highly weighted predictors (eg, phrases such as “wish proceed” and “take operative”) that are not inherently clinical and may reflect aspects of documentation style or templated language rather than surgical technique. This suggests that the model may be partially leveraging institution- or surgeon-specific phrasing patterns. Although variability in note authorship and evolving templates may reduce this effect over time, it highlights a potential limitation related to generalizability, particularly when applying the model to external datasets with different documentation conventions. Training on notes from other institutions will likely be most effective in resolving this issue.

Third, the augmentation of the training data using LLM-generated synthetic notes for procedure subclassification is another potential source of overfitting. Three examples each of cleft lip major revision, cleft lip minor revision, and ABG revision were provided for synthetic data generation. Although the effort was made to select diverse techniques and note styles for these examples, these synthetic notes may, nonetheless, be most similar to the examples provided. In this pilot project, synthetic notes were necessary to explore the feasibility of subclassification for rare procedures. In the future, we will solicit “real-world” operative notes in these categories from across the ACCQUIREnet.

Fourth, our secondary and tertiary classifiers were developed in isolation, given “ground-truth” labeling of preceding classifications. In reality, the hierarchical classification system will need to identify primary, secondary, and tertiary classifications in sequence. Errors in primary classification could permeate into subsequent steps. Therefore, before putting such a system into production, it will be necessary to assess cumulative system accuracy.

Fifth, we excluded a small subset of operative notes that could not be parsed due to missing or nonstandard headers. While this may introduce a minor degree of survivorship bias toward more consistently formatted notes, the proportion excluded was small and unlikely to meaningfully impact overall findings. Future work could incorporate more flexible parsing approaches to further improve robustness to formatting variability.

Sixth, the content validity of the synthetic operative notes was appraised by a senior cleft and craniofacial surgeon (ACA) who is also the corresponding author and lead investigator of this study. As such, this review was internal rather than independent, and the possibility of confirmation bias cannot be excluded. In the future, an independent, blinded clinical review by surgeons unaffiliated with the study would provide stronger evidence of the synthetic notes’ similarity to real-world operative documentation.

Seventh, the ground-truth labels used to train and evaluate all classifiers were generated by a single experienced annotator (CJ), with uncertain cases resolved by a senior surgeon (ACA). Although this annotator has extensive experience in cleft and craniofacial procedural classification, no independent audit was performed on a subset of notes to calculate a formal interrater reliability score. Consequently, the degree of individual annotator bias cannot be quantified, and the possibility that a second annotator would classify some notes differently cannot be excluded. Future work should include independent annotation of a representative subset of notes by a second qualified annotator, with the calculation of a formal interrater reliability statistic (such as Cohen kappa), to better characterize label quality and support confidence in the ground-truth dataset.

### Future Directions

Future directions for this research include prospective validation in a clinical setting, expansion to multi-institutional datasets, and incorporation of additional variables from structured data sources. In addition, it is interesting to note that while it is problematic to go from CPT code to procedural classification, as we have shown in this paper, the opposite direction is easy—to derive an appropriate CPT code from a classified case; therefore, an impactful extension of our work may be to improve CPT coding (and thus billing) using NLP-based classification of operative notes.

### Conclusions

This study demonstrates the feasibility of automatic classification of pediatric craniofacial operative notes across multiple levels of granularity, from primary procedure identification to specific surgical technique recognition. This study paves the way for further development and deployment of such systems, which could significantly reduce the administrative burden involved in surgical research, operations, and quality improvement.

## Abbreviations

ABG: alveolar bone grafting
ACCQUIREnet: Allied Cleft & Craniofacial Quality-Improvement and Research Network
AUC: area under the receiver operating characteristic curve
AUPRC: area under the precision-recall curve
CPT: Current Procedural Terminology
*ICD*: *International Classification of Diseases*
ICHOM: International Consortium for Health Outcomes Measurement
LLM: large language model
NLP: natural language processing
OvR: One-vs-Rest
TF-IDF: term frequency-inverse document frequency
VPI: velopharyngeal insufficiency
